# Predictors of Intraspinal Pressure and Optimal Cord Perfusion Pressure After Traumatic Spinal Cord Injury

**DOI:** 10.1007/s12028-018-0616-7

**Published:** 2018-10-16

**Authors:** Florence R. A. Hogg, Mathew J. Gallagher, Suliang Chen, Argyro Zoumprouli, Marios C. Papadopoulos, Samira Saadoun

**Affiliations:** 1 Academic Neurosurgery Unit, Molecular and Clinical Sciences Institute, St. George’s, University of London, London, SW17 0RE UK; 2 Neuro-intensive Care Units, St. George’s Hospital, London, UK

**Keywords:** Blood pressure, Perfusion pressure, Spinal cord injury, Trauma

## Abstract

**Background/Objectives:**

We recently developed techniques to monitor intraspinal pressure (ISP) and spinal cord perfusion pressure (SCPP) from the injury site to compute the optimum SCPP (SCPP_opt_) in patients with acute traumatic spinal cord injury (TSCI). We hypothesized that ISP and SCPP_opt_ can be predicted using clinical factors instead of ISP monitoring.

**Methods:**

Sixty-four TSCI patients, grades A–C (American spinal injuries association Impairment Scale, AIS), were analyzed. For 24 h after surgery, we monitored ISP and SCPP and computed SCPP_opt_ (SCPP that optimizes pressure reactivity). We studied how well 28 factors correlate with mean ISP or SCPP_opt_ including 7 patient-related, 3 injury-related, 6 management-related, and 12 preoperative MRI-related factors.

**Results:**

All patients underwent surgery to restore normal spinal alignment within 72 h of injury. Fifty-one percentage had U-shaped sPRx versus SCPP curves, thus allowing SCPP_opt_ to be computed. Thirteen percentage, all AIS grade A or B, had no U-shaped sPRx versus SCPP curves. Thirty-six percentage (22/64) had U-shaped sPRx versus SCPP curves, but the SCPP did not reach the minimum of the curve, and thus, an exact SCPP_opt_ could not be calculated. In total 5/28 factors were associated with lower ISP: older age, excess alcohol consumption, nonconus medullaris injury, expansion duroplasty, and less intraoperative bleeding. In a multivariate logistic regression model, these 5 factors predicted ISP as normal or high with 73% accuracy. Only 2/28 factors correlated with lower SCPP_opt_: higher mean ISP and conus medullaris injury. In an ordinal multivariate logistic regression model, these 2 factors predicted SCPP_opt_ as low, medium–low, medium–high, or high with only 42% accuracy. No MRI factors correlated with ISP or SCPP_opt_.

**Conclusions:**

Elevated ISP can be predicted by clinical factors. Modifiable factors that may lower ISP are: reducing surgical bleeding and performing expansion duroplasty. No factors accurately predict SCPP_opt_; thus, invasive monitoring remains the only way to estimate SCPP_opt_.

**Electronic supplementary material:**

The online version of this article (10.1007/s12028-018-0616-7) contains supplementary material, which is available to authorized users.

## Introduction

Traumatic spinal cord injury (TSCI) is a catastrophic condition: Over a third of patients do not recover sensation or voluntary movement below the injury [1]. No treatment has been proved to improve outcome [2], and therefore, patient management remains variable [3]. To guide the management of patients with acute, severe TSCI in the intensive care unit, we introduced intraspinal pressure (ISP) monitoring from the injury site [4, 5]. The technique is safe [6] and allows us to compute the spinal cord perfusion pressure (SCPP) as mean arterial pressure (MAP) minus ISP and the spinal pressure reactivity index (sPRx) as the running correlation coefficient between ISP and MAP. sPRx measures the pressure reactivity of the vascular bed, i.e., autoregulation. sPRx ≤ 0 indicates intact vascular pressure reactivity, whereas sPRx > 0 indicates deficient vascular pressure reactivity. At low SCPP (hypo-perfusion) and high SCPP (hyper-perfusion), autoregulation is impaired (i.e., high sPRx) [7–9]. Thus, the sPRx versus SCPP plot is U-shaped and the optimum SCPP (SCPP_opt_) is the minimum of the curve. The U-shaped curve is not always present and may vary throughout the period of monitoring; when computed in patients with head injury or spinal cord injury, a U-shaped sPRx versus SCPP relation is present approximately 50% of the time. ISP, SCPP, SCPP_opt_, and sPRx for TSCI are, respectively, analogous to intracranial pressure (ICP), cerebral perfusion pressure (CPP), optimum CPP (CPP_opt_), and cerebrovascular pressure reactivity (PRx) for traumatic brain injury (TBI) [10].

TSCI patients with mean SCPP ≈ SCPP_opt_ more often improve their AIS (American spinal injuries association Impairment Scale) grade than patients with SCPP very different from SCPP_opt_ [8]. Thus, targeting the SCPP_opt_ could form the basis for individualizing the treatment of TSCI in a physiologically meaningful manner. When compared with the current guideline of maintaining MAP at 85–95 mmHg in all TSCI patients [11], the concept of individualized management, based on SCPP_opt_, represents a paradigm shift. A universal MAP target is inadequate because TSCI patients have different ISPs and because the SCPP_opt_ varies widely between patients [4, 5, 8]. The aim here is to identify clinical and magnetic resonance imaging (MRI) features for predicting ISP and SCPP_opt_ in each TSCI patient. The ability to estimate ISP and SCPP_opt_ noninvasively would be a major advance by allowing doctors to calculate a target MAP for each TSCI patient without ISP monitoring.

## Materials and Methods

### Institutional Approvals

Patients were recruited as part of the Injured Spinal Cord Pressure Evaluation (ISCoPE) study (ClinicalTrials.gov as NCT02721615). Approvals for ISCoPE were obtained from the St George’s, University of London Joint Research Office and the National Research Ethics Service London–St Giles Committee (No. 10/H0807/23).

### Patient Recruitment

We include all (consecutive) TSCI patients recruited between October 2010 and December 2017. Inclusion criteria are: (1) severe TSCI defined as AIS grade A, B, or C; (2) age 18–70 years; (3) timing between TSCI and surgery within 72 h. Exclusion criteria are: (1) patient unable to consent; (2) other major comorbidities; (3) penetrating TSCI.

### Surgery and ISP Monitoring

Surgical decompression and spinal instrumentation were performed based on patient requirements and surgeon preference. Some patients also had duroplasty as described [12]. During surgery, an ISP probe (Codman Microsensor Transducer^®^, Depuy Synthes, Leeds, UK) was placed intradurally on the surface of the injured cord at the site of maximal cord swelling. The dural opening was sutured and supplemented with fibrin glue (Tisseel^®^, Baxter, UK). The ISP probe was connected to a Codman ICP box linked via a ML221 amplifier to a PowerLab running LabChart v.7.3.5 (AD Instruments, Oxford, UK). Arterial blood pressure was recorded from a radial artery catheter connected to the Philips Intellivue MX800 bedside monitoring system (Philips, Guildford, UK), and in turn connected to the PowerLab system. ISP and arterial blood pressure signals were sampled at 1 kHz for 24 h after surgery. When the spinal cord is swollen and compressed against the dura, the subdural pressure (i.e., ISP) and intraparenchymal pressure at the injury site are the same. In this case, ISP is higher than cerebrospinal fluid (CSF) pressure above or below the injury site—details are given elsewhere [5, 6, 13–15].

### ISP, SCPP, and SCPP_opt_

**Fig. 1:**
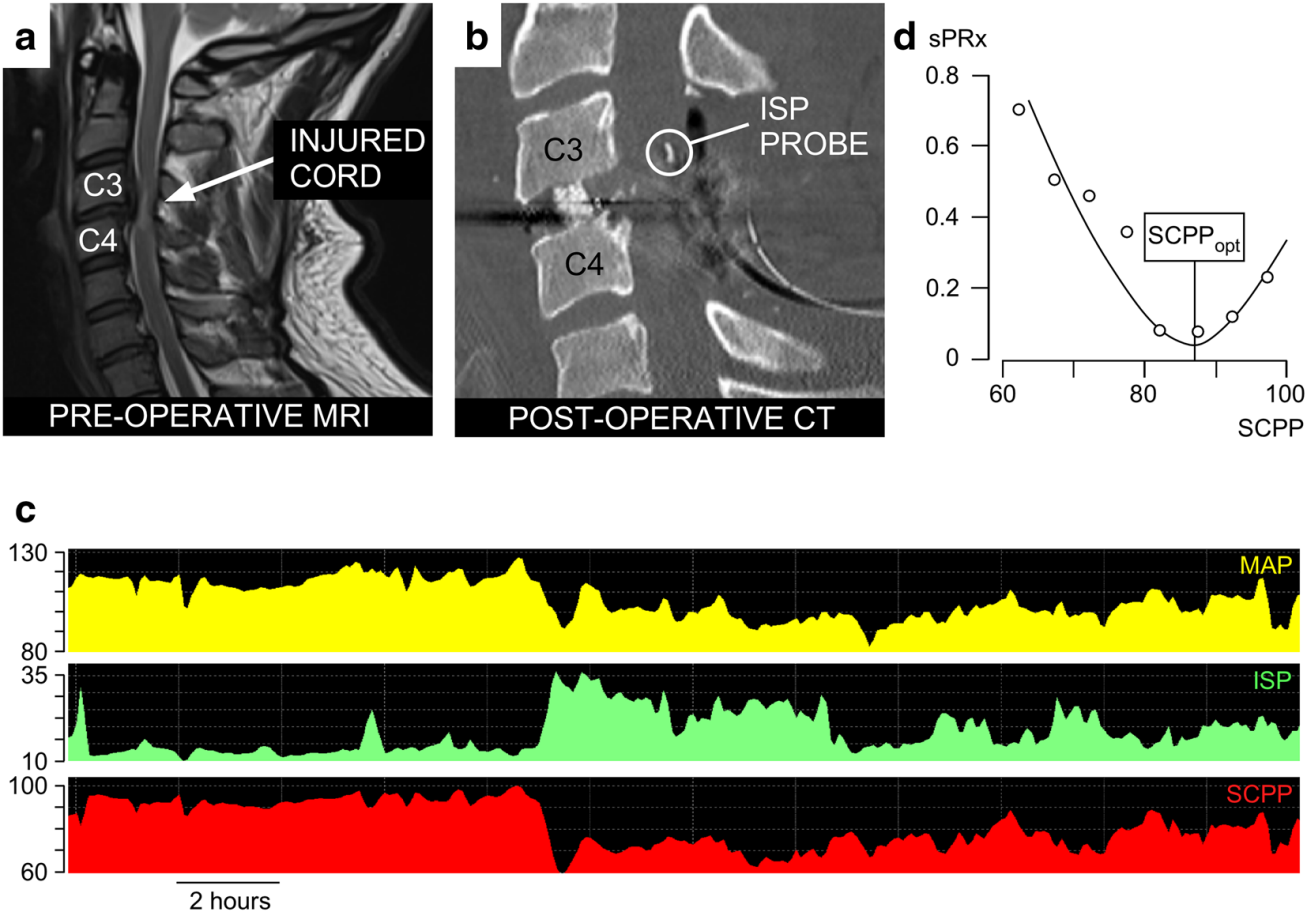
ISP monitoring technique. **a** Preoperative MRI of a 37-year-old male patient with TSCI AIS grade C at C3/4. **b** Postoperative CT of same patient showing C3/4 anterior cervical cage, posterior C3/4 laminectomies, and ISP probe (circled). **c** MAP, ISP SCPP signals. **d** sPRx versus SCPP plot. Minimum is SCPP_opt_

We processed signals using ICM + to compute SCPP (as ISP minus MAP) and sPRx (as the running correlation coefficient between ISP and MAP). Microsoft Excel was used to produce sPRx versus SCPP plots and estimate the SCPP_opt_ as the minimum of the curve. We used the ISP and MAP signals recorded over the 24 h after surgery to compute the average ISP, the average SCPP, as well as the overall SCPP_opt_. The ISP monitoring setup is illustrated in Fig. 1. We chose the first 24 h of ISP monitoring, because these are the closest to the timing of the preoperative MRI that was used to determine the imaging features. Also, because ISP was monitored for different periods in different patients, we only included the first 24 h to standardize the duration of monitoring. We arbitrarily defined ISP as high if > 20 mmHg and normal if ≤ 20 mmHg based on our previous ISP analysis in patients with TSCI versus controls [5]. The study protocol intentionally did not set ISP or SCPP targets, and the ethical approval was to monitor ISP and SCPP, but not use these values to alter management. This arrangement allowed us to study the injured cord over a wide range of ISPs and SCPPs. MAP was managed at the discretion of the consultant intensive care physician. In general, MAP 80–100 mmHg was maintained for a week after TSCI.

### Clinical Features

A total of 28 factors were collected for each patient as follows:

1. Age,
2. Sex,
3. Smoking,
4. Hypertension,
5. Diabetes,
6. History of excess alcohol consumption,
7. Body mass index,
8. Level of injury, i.e., cervical/thoracic/conus,
9. AIS grade on admission,
10. Primary survey MAP,
11. Hours from injury to surgery,
12. Duration of surgery,
13. Intraoperative blood loss,
14. Extent of decompression i.e., spinal alignment/spinal alignment + laminectomy/spinal alignment + laminectomy + duroplasty,
15. Surgical approach, i.e., posterior/anterior + posterior,
16. Mean MAP during surgery,
17. Epidural hematoma,
18. Intraparenchymal hematoma,
19. Cord transection,
20. Number of sagittal inter-vertebral levels with no CSF signal [16],
21. Injury level average spinal cord occupation rate: SCOR [16],
22. Brain and spinal injury center score BASIC [17],
23. Sagittal grade 1–4 [18],
24. Longitudinal extent of T2 signal abnormality [19],
25. Maximum spinal cord compression MSCC [20, 21],
26. Maximum canal compromise MCC [20],
27. Average normal cord in injured cord [22],
28. Average % cord signal change at injured level [16].

Detailed definitions of the 28 factors are given in the Supplement. Data regarding the 28 factors were collected without knowledge of the ISP, SCPP, and SCPP_opt_ data.

### Statistical Analysis

Analyses were carried out with XLStat Biomed (v.18.07, Addinsoft, New York, USA). For univariate analyses, we used Mann–Whitney U or Wilcoxon rank sum with post hoc Dunn to investigate differences in ISP or SCPP_opt_ between the factors. In the multivariate logistic regression analysis for ISP, we chose the best model based on likelihood by including factors with *P *< 0.1 and removing factors already in the model if *P *> 0.2 when a new factor is added. In the multivariate ordinal logistic regression analysis for SCPP_opt_, we chose a stepwise forward model based on the Wald criterion; the model enters factors with *P *< 0.1 and removes factors already in the model if *P *> 0.2 when a new factor is added. Correlations between two factors were quantified with the Spearman’s rank correlation coefficient “*ρ*”.

## Results

### Patient Characteristics

Table 1 provides information on the demographic characteristics of the 64 patients. Most are young, with 80% < 60 years old. Males outnumber females by 3.2:1. Cervical TSCIs are more common than thoracic or conus at 52 versus 34 versus 14%. Most patients had neurologically complete TSCI on admission, i.e., 67% were ASIA grade A. Fifty-one percentage (34/64) had U-shaped sPRx versus SCPP curves for the first 24 h after surgery, thus allowing SCPP_opt_ to be computed. Mean SCPP_opt_ for all patients was 74 mmHg (range 48–103). Thirteen percentage (8/64), all AIS grade A or B, had no U-shaped sPRx versus SCPP curve for the first 24 h after surgery. The remaining 36% (22/64) patients appeared to have U-shaped sPRx versus SCPP curves, but the range of SCPPs did not reach the minimum, and thus, exact SCPP_opt_ could not be calculated.

**Table 1:** Patient demographic characteristics

Characteristic	Value
Patients (total no.)	64
Age in years (mean, range)	42 (19–70)
Sex (m:f)	49:15
Body mass index^a^ (no of patients: < 18.5:18.5–24.9:25–29.9: > 30)	2:30:15:16
Admission AIS grade (no. of patients A:B:C)	43:8:13
Level of injury (no. of patients ce:th:co)	33:22:9
Injury to surgery (hours mean, range)	39.27 (9–72)
Surgical approach (no. of patients post:ant + post)	54:10
Decompression (no. of patients sp-al:sp-al + lami:sp-al + lami + duro)	9:46:9

*AIS* American spinal injuries association Impairment Scale; *ant* anterior; *ce* cervical; *co* conus; *duro* duroplasty; *f* female; *lami* laminectomy; *m* male; *post* posterior; *sp*-*al* spinal alignment; *th* thoracic

^a^Data missing in one patient

### Predictors of Mean ISP

**Fig. 2:**
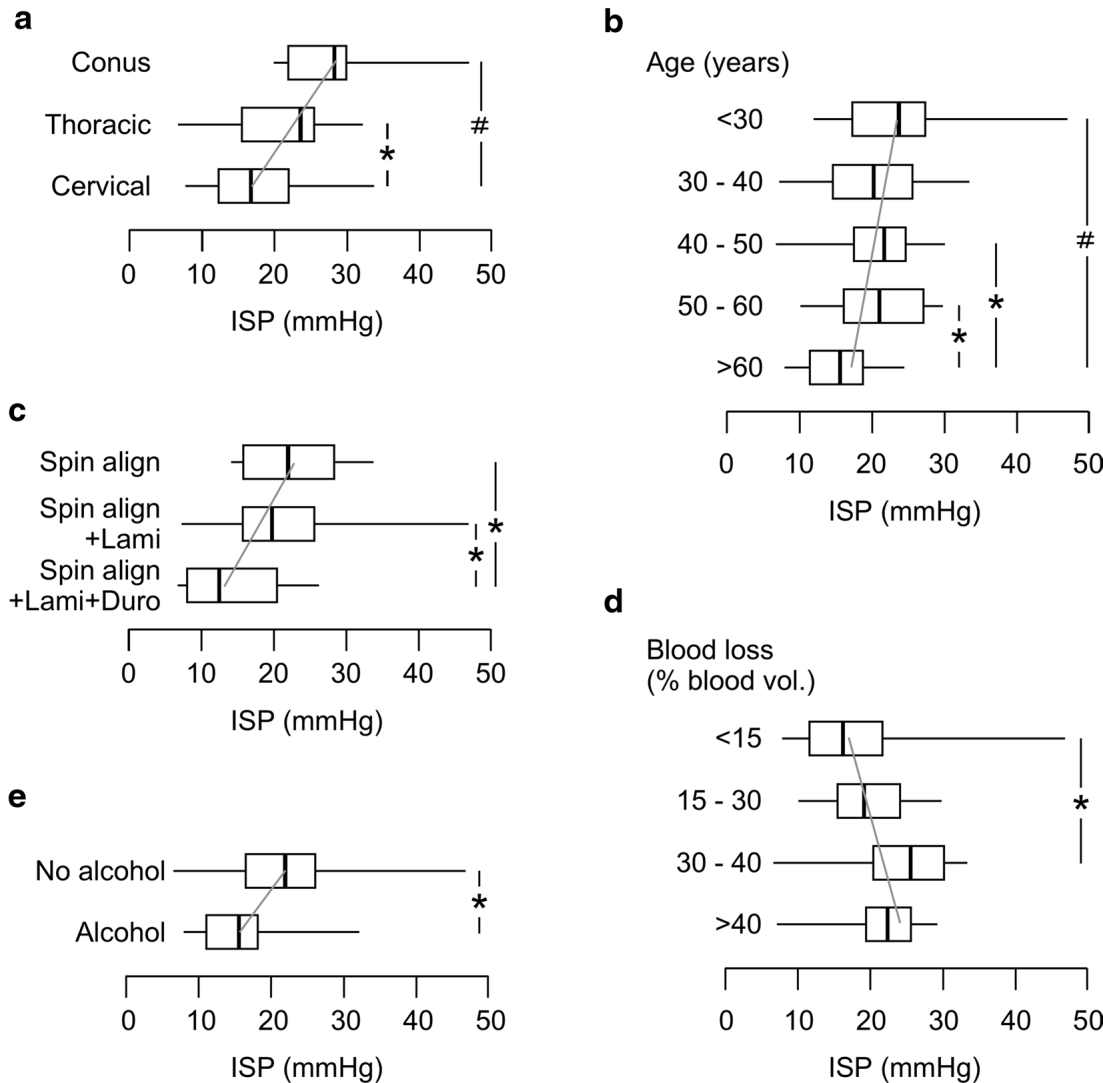
Factors that correlate with mean ISP. **a** Level of spinal cord injury (cervical, thoracic, conus). **b** Age group in years (< 30, 30 – 40, 40 – 50, 50 – 60, > 60). **c** Extent of decompression (Spinal Alignment, Spinal Alignment + Laminectomy, Spinal Alignment + Laminectomy + Duroplasty). **d** Intraoperative blood loss as  % of total blood volume (< 15%, 15 – 30%, 30 – 40%, > 40%). **e** Excess alcohol consumption. Box plots show median, upper and lower quartiles, minimum and maximum. Gray trend line. *P *< 0.05*, 0.005^#^

In univariate analysis, only 5/28 characteristics that were examined correlated with ISP. The 5 characteristics that were associated with higher mean ISP are no excess alcohol consumption, lower cord injury, not having a duroplasty, younger age, and more intraoperative blood loss (Fig. 2). Interestingly, none of the 12 MRI characteristics that we investigated correlated with mean ISP. A multivariate logistic regression model based on these 5 characteristics could classify ISP as normal (< 20 mmHg) versus high (≥ 20 mmHg) with 73% accuracy (Table 2). In this multivariate model, which takes into account interactions between the characteristics, no excess alcohol consumption (odds ratio 9.6, 95% CI 1.2–74.9), lower injury level (odds ratio cervical to thoracic to conus 3.3, 95% CI 1.1–10.3), no expansion duroplasty (odds ratio 3.2, 95% CI 1.1–12.3), and younger age (odds ratio per decade 1.5, 95% CI 1.1–2.4) remained significant (each at *P* < 0.05) predictors of high ISP. Intraoperative blood loss was no longer significant because of collinearity with the level of injury (*ρ* = 0.59), i.e., more blood loss when operating lower down the spine.

**Table 2:** Confusion matrix of multivariate logistic regression model for predicting mean ISP for the first 24 h after surgery

	Normal ISP (predicted)	High ISP (predicted)	Total	% Correct
Normal ISP (actual)	25	8	33	77.8
High ISP (actual)	9	21	30	70.0
Total	34	29	63^a^	73.0

Classifier based on five factors: age group (< 30, 30–40, 40–50, 50–60, > 60), alcohol consumption (yes/no), spinal level of injury (cervical/thoracic/conus), extent of decompression (spinal alignment, spinal alignment + laminectomy, spinal alignment + laminectomy + duroplasty), and intraoperative hemorrhage (as  % of circulating volume: < 15, 15–30, 30–40, > 40). Area under receiver operating characteristic curve = 0.84

*ISP* intraspinal pressure

^a^1/64 patients omitted because of missing intraoperative data

### Predictors of Mean 24-h SCPP_opt_

**Fig. 3:**
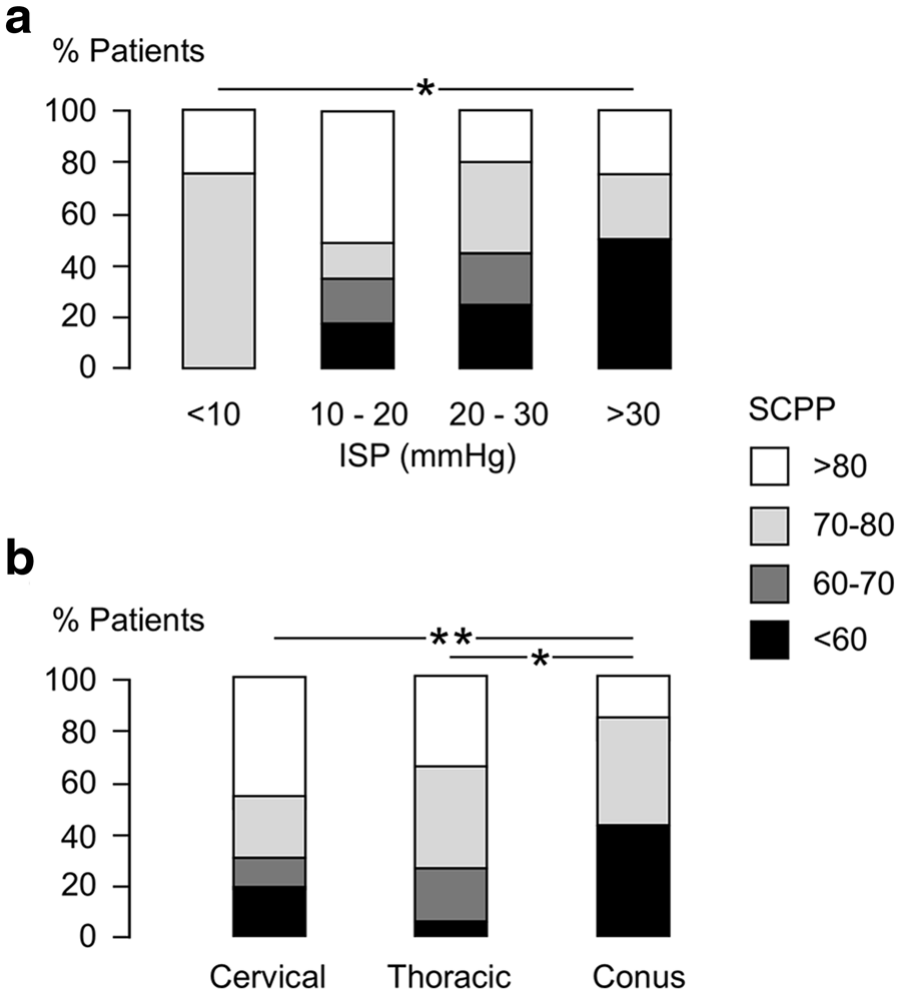
Factors that correlate with SCPP_opt_. **a** Mean ISP group (< 10, 10–20, 20–30, > 30 mmHg). **b** Level of spinal cord injury (cervical, thoracic, conus). SCPP_opt_ grouped as < 60, 60–70, 70–80, > 80 mmHg. *P *< 0.05*, 0.01**

Each patient was assigned to a SCPP_opt_ group as low (< 60 mmHg), low–medium (60–70 mmHg), high–medium (70–80 mmHg), or high (> 80 mmHg). This allowed us to analyze 51 patients in total, by including not only the 34 patients who had exact SCPP_opt_, but also 8 patients with SCPP_opt_ > 80 mmHg and 9 patients with SCPP_opt_ < 60 mmHg. In univariate analysis, only 2/29 factors that were examined correlated with lower mean SCPP_opt_: higher mean 24-h ISP and lower spinal level of injury (Fig. 3a, b). These two factors (level of spinal cord injury and mean 24-h ISP) were correlated (*ρ* = 0.49). Interestingly, again none of the 12 MRI factors correlated with mean SCPP_opt_. An ordinal logistic regression model based on these two factors could classify SCPP_opt_ as low, low–medium, high–medium, or high with only 42% accuracy (Table 3).

**Table 3:** Confusion matrix of multivariate ordinal logistic regression model for predicting SCPP_opt_ for the first 24 h after surgery

SCPP_opt_ mmHg	Predicted					
	<60	60–70	70–80	>80	Total	% Correct
*Actual*						
<60	4	0	1	5	10	40.0
60–70	3	0	3	3	9	0.0
70–80	1	0	6	7	14	42.9
>80	1	0	2	15	18	83.3
*Total*						
	8	0	6	37	51^a^	41.6

Classifier based on two factors: mean 24 h and spinal level of injury (cervical/thoracic/conus)

*SCPP* spinal cord perfusion pressure

^a^13/64 patients omitted because SCPP_opt_ could not be binned into one of these groups (< 60, 60–70, 70–80, > 80)

## Discussion

In this study we have shown that after a TSCI clinical features may be used to predict whether ISP is elevated, but clinical features cannot accurately predict SCPP_opt_. Risk factors for high ISP include younger age, conus injury, no excessive alcohol intake, no duroplasty, and large intraoperative blood loss. We also showed that patients with conus injuries and those with high ISP have low SCPP_opt_ and that some patients with severe TSCI have no computable SCPP_opt_. There were no MRI features associated with ISP or SCPP_opt_.

Clinical features that correlate with ISP may shed light on the pathophysiology of TSCI. The factor that protects the most from elevated ISP is history of excess alcohol consumption documented in the patient notes. The underlying mechanism may be cord atrophy, i.e., more CSF space to accommodate the swollen cord, analogous to TBI where alcohol-related brain atrophy might protect from elevated ICP [23–25]. Expansion duroplasty and the level of injury have comparable protective effect on ISP. We already showed that duroplasty significantly reduces ISP after TSCI by enlarging the CSF space around the injured cord [12]. The higher ISP with conus medullaris injuries is likely related to the reduced CSF space around the conus medullaris compared with the larger CSF space higher up the spine [26]. The next factor that correlates with lower ISP is older age, perhaps because of cord atrophy in older patients, analogous to age-related brain atrophy [27, 28]. Higher ISP in patients with more intraoperative bleeding may be due to the level of TSCI rather than the bleeding per se, based on the positive correlation between surgical blood loss and level of TSCI. Alternatively, intraoperative fluid resuscitation may play a role, with larger fluid volumes administered exacerbating the edema at the injury site. The unifying theme here is that ISP after TSCI is determined by the relative dimensions of the cord at the injury site compared with the surrounding CSF space. Larger CSF space (cord atrophy, cervicothoracic injuries, duroplasty) is associated with lower ISP.

We showed that SCPP_opt_ is lower in patients with higher ISP or conus TSCI. Though high SCPP is beneficial by reducing ischemia, these observations suggest that as the spinal cord becomes more compressed, high SCPP may become detrimental. The mechanism might be blood pressure induced local steal, a phenomenon whereby increasing the MAP when the cord is swollen causes a “paradoxical” decrease in blood flow at the injury site [29]. Knowledge of SCPP_opt_ is clinically important to reduce secondary cord damage by hypo- or hyper-perfusion. Since clinical features do not accurately predict SCPP_opt_, invasive ISP monitoring remains the only way of determining SCPP_opt_. A recent study showed that SCPP can be calculated by monitoring CSF pressure using a lumbar catheter and that SCPP measured in this way better correlates with outcome than MAP [30]. Monitoring CSF pressure is less invasive than monitoring ISP. However, the relation between SCPP measured by ISP monitoring and by lumbar drain is unclear. It is also unclear whether CSF pressure can be used to compute sPRx and SCPP_opt_.

An unanticipated finding is that no MRI features correlated with ISP or SCPP_opt_. This may be because we used preoperative MRIs, performed when the spinal anatomy was abnormal. Surgery restores the normal spinal alignment, thus causing major anatomical changes at the injury site. MRI performed immediately after surgery may thus be more informative for estimating ISP and SCPP_opt_ than preoperative MRIs. Earlier studies support the notion preoperative MRI features only weakly correlate with outcome, and in general, postoperative MRIs are more informative [31–33]. The take-home message is that preoperative MRIs cannot be relied upon to estimate the degree of cord compression or the optimal MAP to target after surgery.

Our study has limitations. ISP monitoring is an invasive technique, and currently, ours is the only unit that performs such monitoring in acute TSCI. Corroboration of our results in other centers is essential. Thus, the concepts of ISP, SCPP, SCPP_opt,_ and sPRx remain theoretical and a randomized study to assess outcomes of patients managed according to their individual injury site physiology rather than applying universal MAP targets is necessary before ISP monitoring becomes definitively recommended. Finally, our cohort of 64 patients is relatively small, and thus, weaker associations between the 28 clinical features investigated here ISP and SCPP_opt_ might be missed.

## Conclusions

To conclude, our study supports the notion that ISP is determined by the size of the CSF space surrounding the injured cord. In other words, after TSCI, the cord swells and is compressed by the dura [34], thus, factors that increase the surrounding CSF space such as expansion duraplasty may be beneficial [12]. Based on our data, we urge caution when increasing the MAP, without knowing the SCPP_opt_, in patients whose injured cord is very compressed, i.e., high ISP. The individualized U-shaped curves suggest that SCPP > SCPP_opt_ may be detrimental. Hyper-perfusing injured cord is associated with more deranged injury site metabolism [35]. In TBI patients, hyper-perfusion of the brain is associated with worse neurological outcome [36], although this finding has not yet been replicated in TSCI (due to insufficient number of TSCI patients exceeding the SCPP_opt_), the same trend may apply [8]. To date, the only way to avoid hyper-perfusing the injured cord is by determining the SCPP_opt_ by ISP monitoring.

At present there is no consensus about the correct paradigm for managing acute TSCI [2, 3, 37, 38] although there is growing evidence and support for a randomized trial to assess whether novel treatments such as invasive ISP monitoring, SCPP optimization, and expansion duroplasty improve outcomes [39].

## Electronic supplementary material

Below is the link to the electronic supplementary material.

Supplementary material 1 (PDF 1101 kb)
