# Supplementary material for: Predictors of Intraspinal Pressure and Optimal Cord Perfusion Pressure After Traumatic Spinal Cord Injury
Source: Neurocrit Care. 2018 Oct 16;30(2):421–8. doi: 10.1007/s12028-018-0616-7 (PMC6420421; doi:10.1007/s12028-018-0616-7)
Supplement: Supplementary file 1 — Supplementary material 1 (PDF 1101 kb) [file 12028_2018_616_MOESM1_ESM.pdf]

SUPPLEMENT

The 28 Factors Assessed

7 Patient Related Factors

**Age**	
**Sex**	
**Smoking**	Any history of smoking, either past or present
**Hypertension**	Pre-existing diagnosis of hypertension, identified in medical / GP records
**Diabetes Mellitus**	Any diagnosis of type I or II diabetes mellitus identified in medical / GP records
**Excess alcohol consumption**	As recorded in medical / GP records
**Body Mass Index**	Patients weight on admission to hospital (kg)/height (metres) [REF 2]

3 Injury Related Factors

**Level of Injury**	Classified as: cervical (C1-7), thoracic (T1-T11) and conus (T12-L1)
**AIS grade on admission**	American spinal injury association Impairment Scale [REF 1] assessed by neurosurgical team
**Primary Survey MAP**	Lowest MAP (1/3 systolic + 2/3 diastolic) recorded in trauma admission notes.

6 Management Related Factors

**Hours from injury to surgery**	
**Duration of surgery**	As documented in operative notes
**Intraoperative blood loss**	Blood vol. =75 ml/kg. Blood loss graded 1–4 as % of blood vol. (1:<15%, 2:15–30%, 3:30 –40%, 4:>40%) [REF 2]
**Extent of decompression**	Spinal alignment / Spinal alignment and laminectomy / Spinal alignment and laminectomy and duroplasty
**Surgical Approach**	Posterior / Posterior and anterior
**Mean MAP during surgery**	Average of the mean arterial pressures (1/3 systolic + 2/3 diastolic) recorded in anesthetic operative notes.

12 Pre-operative MRI related Factors

**Epidural hematoma**	Assessed on T1 and T2 sagittal and axial images
**Intraparenchymal hematoma**	Assessed on T1 and T2 sagittal and axial images
**Cord transection**	T2 sagittal. Cord obliterated; complete translocation of anterior and posterior cord margins cranial and caudal to injury.
**No. of sagittal inter-vertebral levels with no CSF signal**	Graded qualitatively on T2 mid-sagittal imaging. Pattern defined as normal (CSF visible around the cord), partial (partial effacement of CSF around the cord) and complete (total loss of CSF signal around the cord) (Supp. Fig. 1A)

**Inj. Level Ave. Sp. Cord Occupation Rate: SCOR**	Mid Sagittal Cord Diameter / Mid sagittal dural sac diameter *100<br>Calculated at each vertebral and intravertebral level throughout the region of spinal injury. (Supp. Fig. 1A)
**Maximum Spinal Cord Compression (MSCC)**	Diameter of spinal cord at maximal compromise / average diameter of spinal cord above & below x100% (Supp. Fig. 1A)
**Maximal Canal Compromise (MCC)**	Diameter of canal at maximal compromise / average diameter of canal above & below injury x100% (Supp. Fig. 1A)
**Sagittal extent of T2 cord signal abnormality**	Measured in mm on mid sagittal T2 weighted imaging (Supp. Fig. 1B)
**Average normal cord in injured cord**	Mid Sagittal T2 Cord diameter – Mid Signal change diameter<br>Based on 'space available for cord' calculation and developed for this analysis (Supp. Fig. 1B)
**% Injured Cord Signal Change**	(mid sagittal diameter of cord signal change / mid sagittal cord diameter) x100<br>Based on 'spinal cord occupation ratio' and developed for this analysis. (Supp. Fig. 1B)
**BASIC Score (0 – 4)**	0: no cord signal abnormality, 1: T2 hyperintensity confined to grey matter, 2: intrameduallary T2 hyperintensity extends beyond grey matter involving some (not all) white matter, 3: T2 hyperintensity involving grey matter and all of the white matter, 4: T2 signal hyperintensity with area of hypointensity representing hemorrhage in cord (Supp. Fig. 2)
**Sagittal Grade**	1: no signal change, 2: single level T2 hyperintensity, 3: >1 vertebral level T2 signal hyperintensity, 4: T2 signal hyperintensity with areas of hypointensity representing haemorrhage (Supp. Fig. 3)

REF 1. Association ASI. American Spinal Injury Association: International Standards for Neurological Classification of Spinal Cord Injury.

REF 2. Gutierrez G, Reines HD, Wulf-Gutierrez ME. Clinical review: hemorrhagic shock. Crit Care 2004;8:373-81.

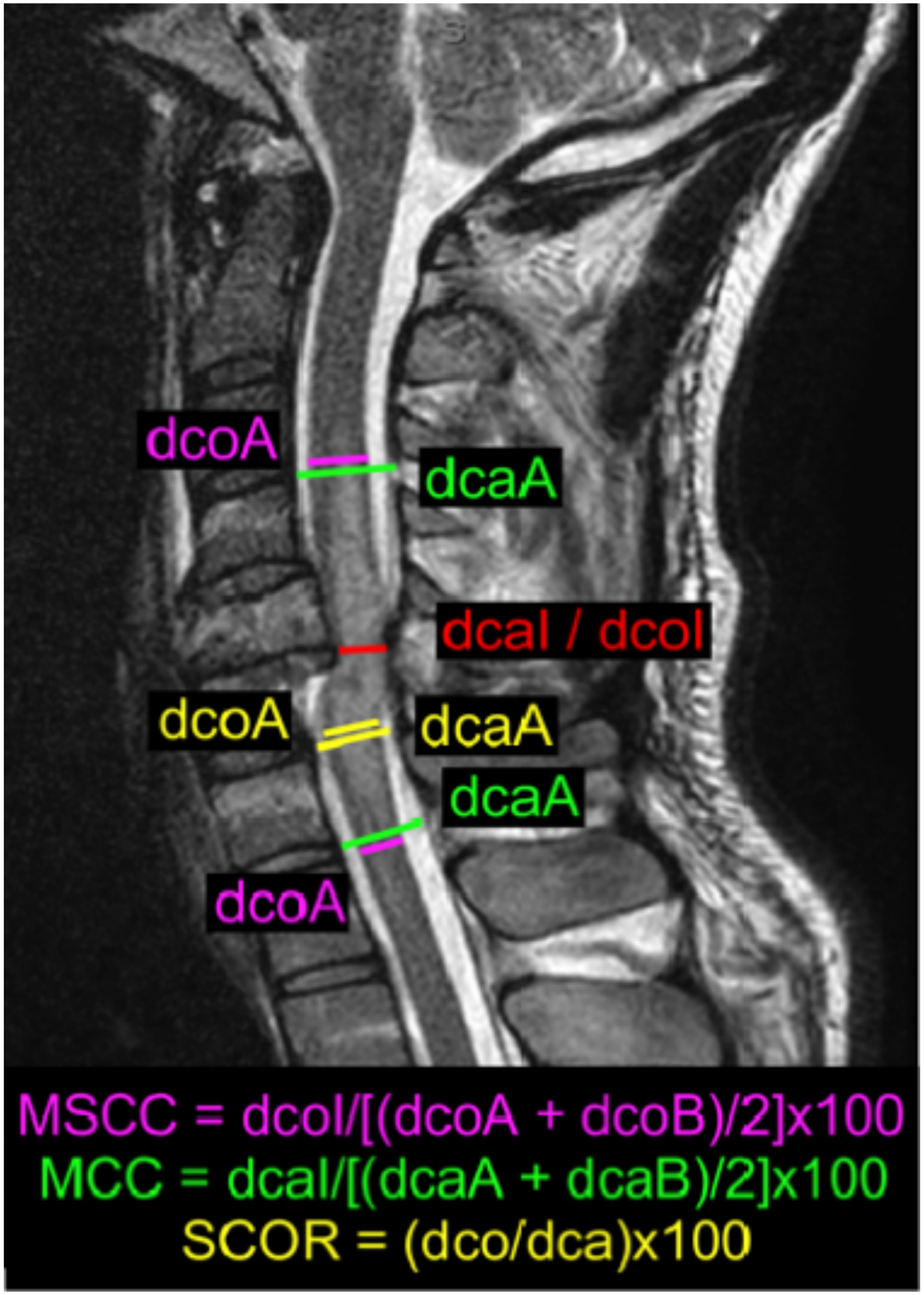

SUPP. FIG. 1A. Spinal Cord injury MRI Grading Systems.

Maximal Spinal Cord Compression (MSCC)

Maximum Canal Compression (MCC)

Spinal Cord Occupation Ratio (SCOR)

dca = diameter of canal

dcaA = diameter of canal above injury

dcaB = diameter of canal below injury

dcaI = diameter of canal at injury level

dco = diameter of cord

dcoA = diameter of cord above injury

dcoB = diameter of cord below injury

dcoI = diameter of cord at level of injury

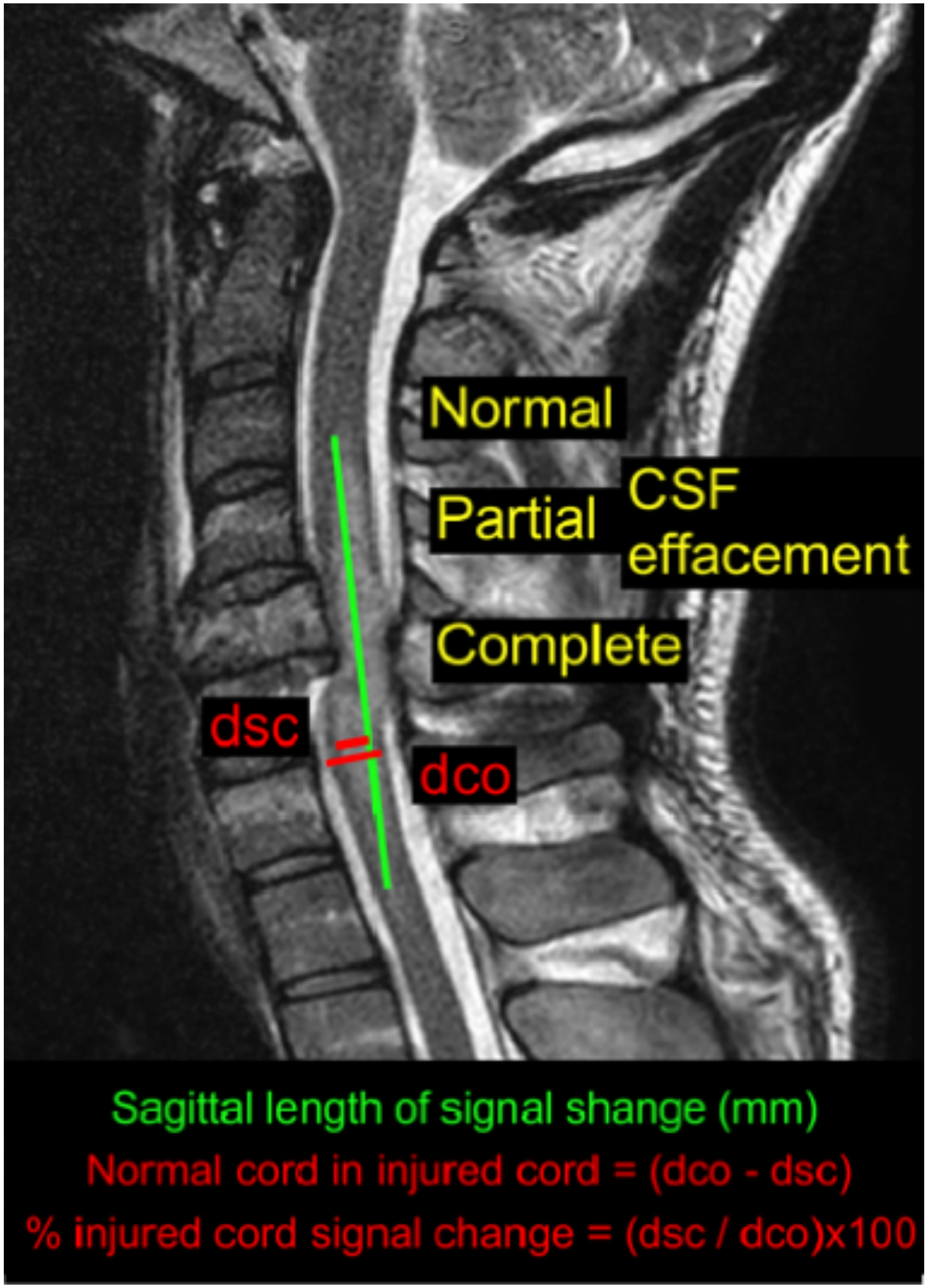

SUPP. FIG. 1B. Spinal Cord injury MRI Grading Systems.

CSF effacement around cord

Sagittal length

% cord signal change

Normal cord in injured cord

dco = diameter of cord

dsc = diameter of cord signal change

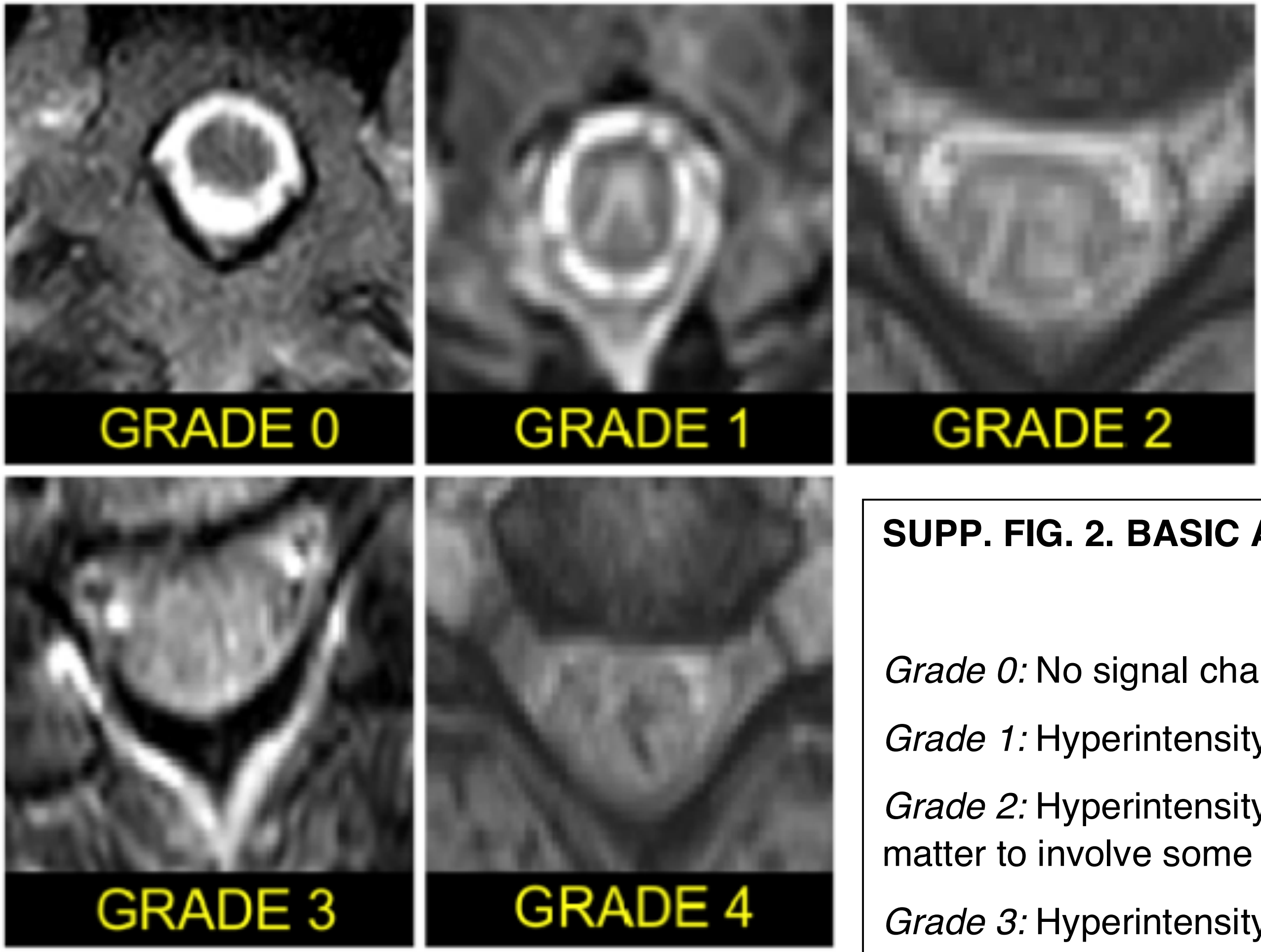

SUPP. FIG. 2. BASIC Axial MRI T2 grading

Grade 0: No signal change

Grade 1: Hyperintensity confined to grey matter

Grade 2: Hyperintensity spreads beyond grey matter to involve some but not all white matter

Grade 3: Hyperintensity involves whole axial cord

Grade 4: Area of hypointensity indicative of hemorrhage

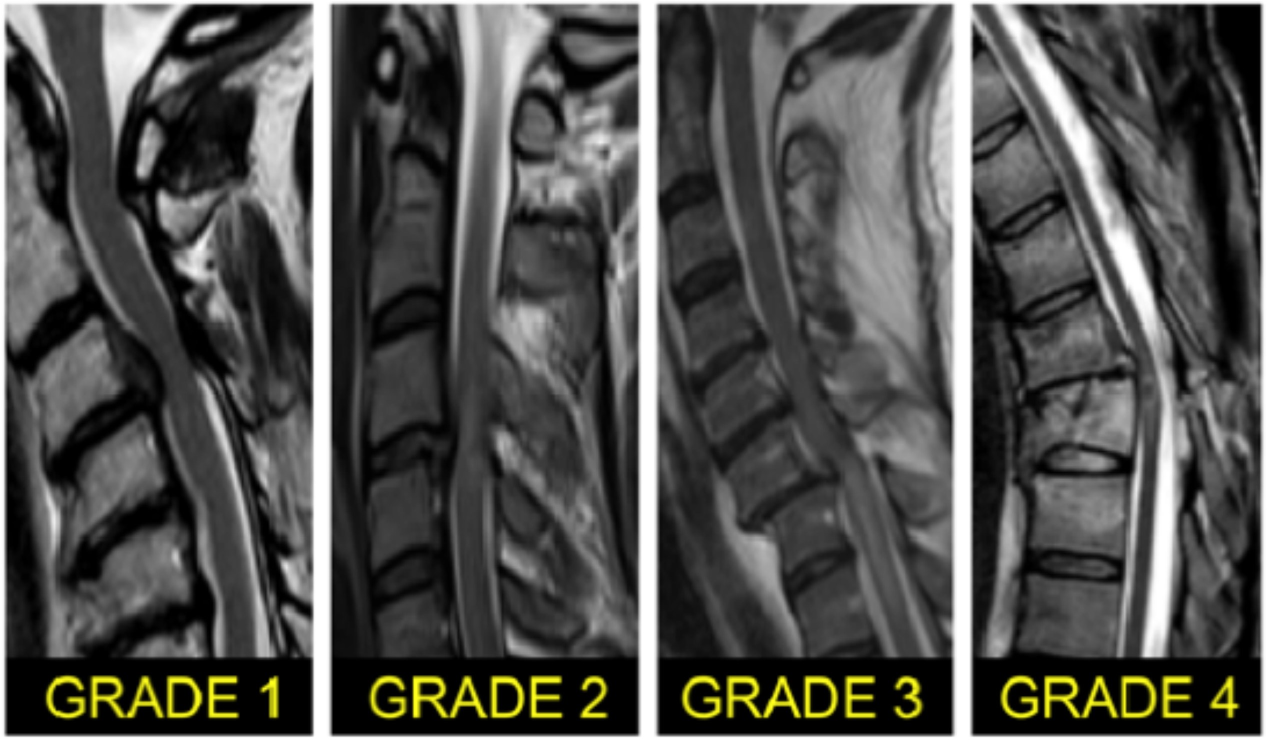

SUPP. FIG. 3. Sagittal grade.

Grade 1: No cord signal change.

Grade 2: T2 hyperintensity confined to only 1 vertebral body.

Grade 3: T2 hyperintensity spanning >1 vertebral body.

Grade 4: Area of hypointensity indicative of hematoma
